# Supplementary material for: Different environmental variables predict body and brain size evolution in Homo
Source: Nat Commun. 2021 Jul 8;12:4116. doi: 10.1038/s41467-021-24290-7 (PMC8266824; doi:10.1038/s41467-021-24290-7)

Supplementary Information

Different environmental variables predict body and brain size evolution in *Homo*

(Nature Communications)

Manuel Will[1], Mario Krapp[2,3], Jay T. Stock[4,5], Andrea Manica[2]

[1] Department of Early Prehistory and Quaternary Ecology, University of Tübingen, Tübingen, Germany

[2] Evolutionary Ecology Group, Department of Zoology, University of Cambridge, Cambridge, United Kingdom

[3] GNS Science, Lower Hutt, New Zealand

[4] Department of Anthropology, Western University, Canada

[5] Department of Archaeology, Max Planck Institute for the Science of Human History, Jena, Germany

Table of contents

Item	Page
Supplementary Table 1	2
Supplementary Table 2	3
Supplementary Table 3	4
Supplementary Note 1	5
Supplementary Figure 1	6
Supplementary Figure 2	7
Supplementary Figure 3	8
Supplementary Figure 4	9
Supplementary Figure 5	10
Supplementary Figure 6	11
Supplementary Figure 7	12
Supplementary Figure 8	13
Supplementary Figure 9	14
Supplementary Figure 10	15
Supplementary Figure 11	16

Supplementary Table 1. Analyses of fossil data for body size and environmental variables based on 1000 randomly thinned datasets (not log-transformed). The median effect sizes (slope) for *LM-TC* and *LM-T*C* are shown with their 95% range (2.5-97.5%) based on the 1000 thinned datasets. Either of the two models is highlighted in bold if it is the best model based on AIC (no highlight for a given variable means that the *LM-T* is better than either of the two). The percentage in [] indicates how often (out of the 1000 randomly thinned datasets) a given model was the best among all models. The R^2 values (with their 95% range) indicate how much more variance in body size can be explained compared to *LM-T*. For reference, the R^2 of *LM-T* is 0.05 (0.02, 0.08).

**Variable/effect**	***LM-TC***	***LM-T*C*: MP *Homo***	***LM-T*C*: Neanderthals**	***LM-T*C*: *Homo sapiens***	**R^2 *LM-TC***	**R^2 *LM-T*C***
**MAT**	**-0.57 (-0.71, -0.42)**	-0.67 (-1.0, -0.32)	-0.82 (-1.2, -0.51)	-0.5 (-0.68, -0.32)	**0.21 (0.14, 0.29) [98%]**	0.22 (0.15, 0.30) [2%]
MAP	-0.0014 (-0.0047, 0.0027)	0.00051 (-0.008, 0.013)	0.009 (0.0014, 0.017)	-0.0048 (-0.0088, -0.00026)	0.05 (0.03, 0.09) [0%]	0.09 (0.04, 0.14) [3%]
**Mean temperature of coldest quarter**	**-0.41 (-0.5, -0.31)**	-0.33 (-0.48, -0.19)	-0.7 (-0.94, -0.45)	-0.4 (-0.51, -0.27)	**0.20 (0.14, 0.27) [99%]**	0.21 (0.14, 0.28) [0%]
Mean precipitation of driest quarter	0.0042 (0.0011, 0.0076)	0.014 (-0.002, 0.026)	0.011 (0.0069, 0.016)	-0.0004 (-0.0045, 0.0039)	0.07 (0.03, 0.11) [3%]	0.10 (0.05, 0.16) [10%]
NPP	-0.009 (-0.015, -0.0022)	-0.0058 (-0.022, 0.014)	0.0067 (-0.011, 0.028)	-0.014 (-0.021, -0.0053)	0.08 (0.04, 0.13) [23%]	0.10 (0.05, 0.17) [10%]
MATvar10	2.6 (-1.9, 7.2)	16.0 (-6.1, 39.0)	5.4 (-12.0, 22.0)	0.15 (-4.2, 4.4)	0.06 (0.03, 0.10) [0%]	0.09 (0.04, 0.17) [15%]
MAPvar10	-1.1 (-2.3, 0.39)	-3.2 (-7.3, 1.1)	1.2 (-1.7, 4.5)	-0.99 (-2.7, 0.87)	0.06 (0.03, 0.11) [5%]	0.09 (0.04, 0.17) [12%]
Mean temperature of coldest quarter (var10)	3.4 (-1.2, 8.5)	13.0 (-2.4, 30.0)	4.7 (-7.7, 16.0)	0.29 (-4.7, 5.8)	0.06 (0.03, 0.11) [3%]	0.09 (0.04, 0.17) [11%]
mean precipitation of driest quarter (var10)	0.33 (-0.36, 1.1)	1.2 (-1.1, 4.0)	1.8 (0.55, 3.3)	-0.44 (-1.4, 0.52)	0.05 (0.03, 0.09) [0%]	0.08 (0.04, 0.14) [3%]
NPPvar10	0.46 (-0.83, 1.8)	-1.8 (-6.8, 3.2)	1.4 (-1.4, 4.4)	0.77 (-0.55, 2.2)	0.05 (0.03, 0.09) [1%]	0.07 (0.04, 0.14) [3%]

Supplementary Table 2. Analyses of fossil data for brain size and environmental variables based on 1000 randomly thinned datasets (not log-transformed). The median effect sizes (slope) of *LM-TC* and *LM-T*C* are shown with their 95% range (2.5-97.5%) based on the 1000 thinned datasets. Either of the two models is highlighted in bold if it is the best model based on AIC (no highlight for a given variable means that the *LM-T* is better than either of the two). The percentage in [] indicates how often (out of the 1000 randomly thinned datasets) a given model was the best among all models. The R^2 values (with their 95% range) indicate how much more variance in brain size can be explained compared to *LM-T*. For reference, the R^2 of *LM-T* is 0.46 (0.42, 0.50).

**Variable/effect**	***LM-TC***	***LM-T*C*:** **MP *Homo***	***LM-T*C*:** **Neanderthals**	***LM-T*C*:** ***Homo sapiens***	**R^2 *LM-TC***	**R^2 *LM-T*C***
MAT	-1.7 (-3.3, -0.28)	-5.3 (-8.5, -2.2)	1.6 (-5.4, 8.7)	-0.65 (-2.5, 1.2)	0.46 (0.43, 0.50) [0%]	0.48 (0.44, 0.51) [0%]
MAP	-0.064 (-0.12, -0.015)	-0.12 (-0.2, -0.046)	-0.17 (-0.45, 0.028)	0.02 (-0.054, 0.099)	0.47 (0.43, 0.51) [10%]	0.49 (0.44, 0.54) [20%]
Mean temperature of coldest quarter	-1.0 (-2.0, -0.21)	-2.7 (-4.4, -1.1)	-3.0 (-7.4, 1.7)	-0.076 (-1.1, 1.0)	0.46 (0.42, 0.50) [0%]	0.47 (0.43, 0.51) [0%]
mean precipitation of driest quarter	-0.022 (-0.079, 0.035)	-0.014 (-0.14, 0.093)	-0.032 (-0.12, 0.05)	-0.018 (-0.11, 0.074)	0.46 (0.42, 0.50) [0%]	0.46 (0.42, 0.50) [0%]
NPP	-0.13 (-0.23, -0.046)	-0.25 (-0.41, -0.11)	-0.3 (-0.77, 0.12)	0.0097 (-0.12, 0.14)	0.48 (0.44, 0.52) [16%]	0.50 (0.45, 0.55) [33%]
MATvar10	12.0 (-63.0, 91.0)	160.0 (14.0, 350.0)	-94.0 (-300.0, 110.0)	-33.0 (-130.0, 56.0)	0.46 (0.42, 0.50) [0%]	0.48 (0.44, 0.54) [17%]
**MAPvar10**	-35.0 (-54.0, -16.0)	-39.0 (-73.0, -9.7)	-51.0 (-130.0, 24.0)	-28.0 (-53.0, -2.4)	0.49 (0.44, 0.55) [71%]	0.50 (0.45, 0.56) [5%]
Mean temperature of coldest quarter (var10)	-22.0 (-89.0, 44.0)	99.0 (-18.0, 240.0)	-110.0 (-260.0, 31.0)	-100.0 (-200.0, -1.0)	0.46 (0.42, 0.50) [0%]	0.49 (0.44, 0.54) [20%]
Mean precipitation of driest quarter (var10)	-16.0 (-28.0, -3.1)	-32.0 (-54.0, -7.2)	-30.0 (-56.0, -2.1)	2.8 (-16.0, 21.0)	0.47 (0.43, 0.51) [10%]	0.49 (0.44, 0.53) [9%]
NPPvar10	-16.0 (-36.0, 4.1)	-14.0 (-55.0, 22.0)	-45.0 (-110.0, 16.0)	-8.0 (-32.0, 17.0)	0.47 (0.43, 0.51) [7%]	0.48 (0.43, 0.52) [3%]

Supplementary Table 3. Hypothetical *strong* effect sizes used in the power analysis for both body and brain size (log and natural units). Listed are the hypothetical *strong* effect sizes. *Medium* and *weak* effects correspond to ½ or ¼ of the *strong* effect. Units for the log-transformed sizes are *% change per climate variable unit*. Natural units are *kg* (body size) or *cm*^{3} (brain size) *per climate variable unit*.

Variable/effect	body (log)	body (natural)	brain (log)	brain (natural)
MAT	0.57	0.35	0.55	7.1
MAP	4.79	2.99	5.19	67.3
Mean temperature of coldest quarter	0.37	0.23	0.36	4.7
Mean precipitation of driest quarter	2.96	1.85	2.89	37.5
NPP	0.02	0.02	0.02	0.3
MATvar10	19.15	11.97	17.66	229.2
MAPvar10	4.41	2.76	3.64	47.2
Mean temperature of coldest quarter (var10)	13.57	8.48	15.44	200.4
Mean precipitation of driest quarter (var10)	4.28	2.67	3.89	50.5
NPPvar10	4.90	3.06	4.16	54.0

Supplementary Note 1. Distribution map of fossil data (brain and body size).

An interactive and scalable map illustrating the geographical for all *Homo* specimens involved in this study can be found via the following URL:

http://www.roceeh.uni-tuebingen.de/maps/brain_body_map/

Citable DOI: 10.5281/zenodo.3660414

In addition to the spatial location of each data point, additional information from Supplementary Data 1 and Supplementary Data 2 are displayed. Sources for all relevant background data (OSM) and meta-data are indicated in the map (bottom of the page).

Supplementary Figure 1. **Power analysis showing the proportion of synthetic datasets (*n*=1000) for which a relationship using the *LM-TC* model is detectable, i.e., ΔAIC for the null model >2.** The color gradient in each panel indicates how many such relationships can be detected within each single synthetic data set, resolved as vertical bands in each panel. Body and brain size are used in their natural units.

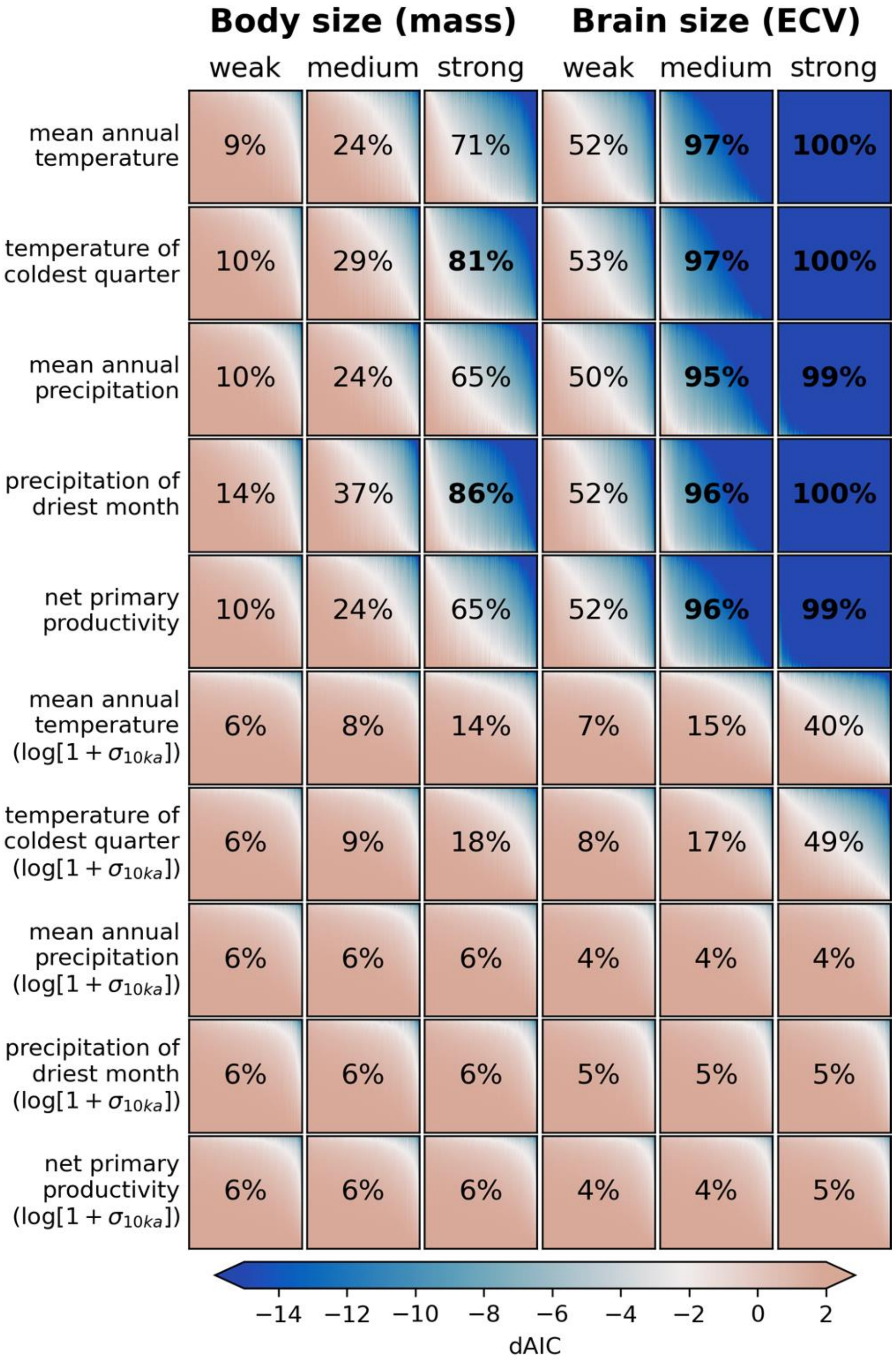

Supplementary Figure 2. Analysis of body size (natural units) fossil data with the *LM-TC* model. Relationships based on *LM-TC* between body size from the fossil data sets and the ten climate variables based on the local climate reconstructions. The shaded band corresponds to the 95-percentile range (2.5-97.5%) of all linear regression lines that have been calculated for the 1000 randomized and thinned samples with the thick line in the centre corresponding to the median (50th-percentile). Each semi-transparent point represents a single fossil record, whereas the opaque points represent a record from a randomly thinned sub-sample. Numbers in brackets indicate the number of fossil records for each taxonomic unit in thinned subsamples.

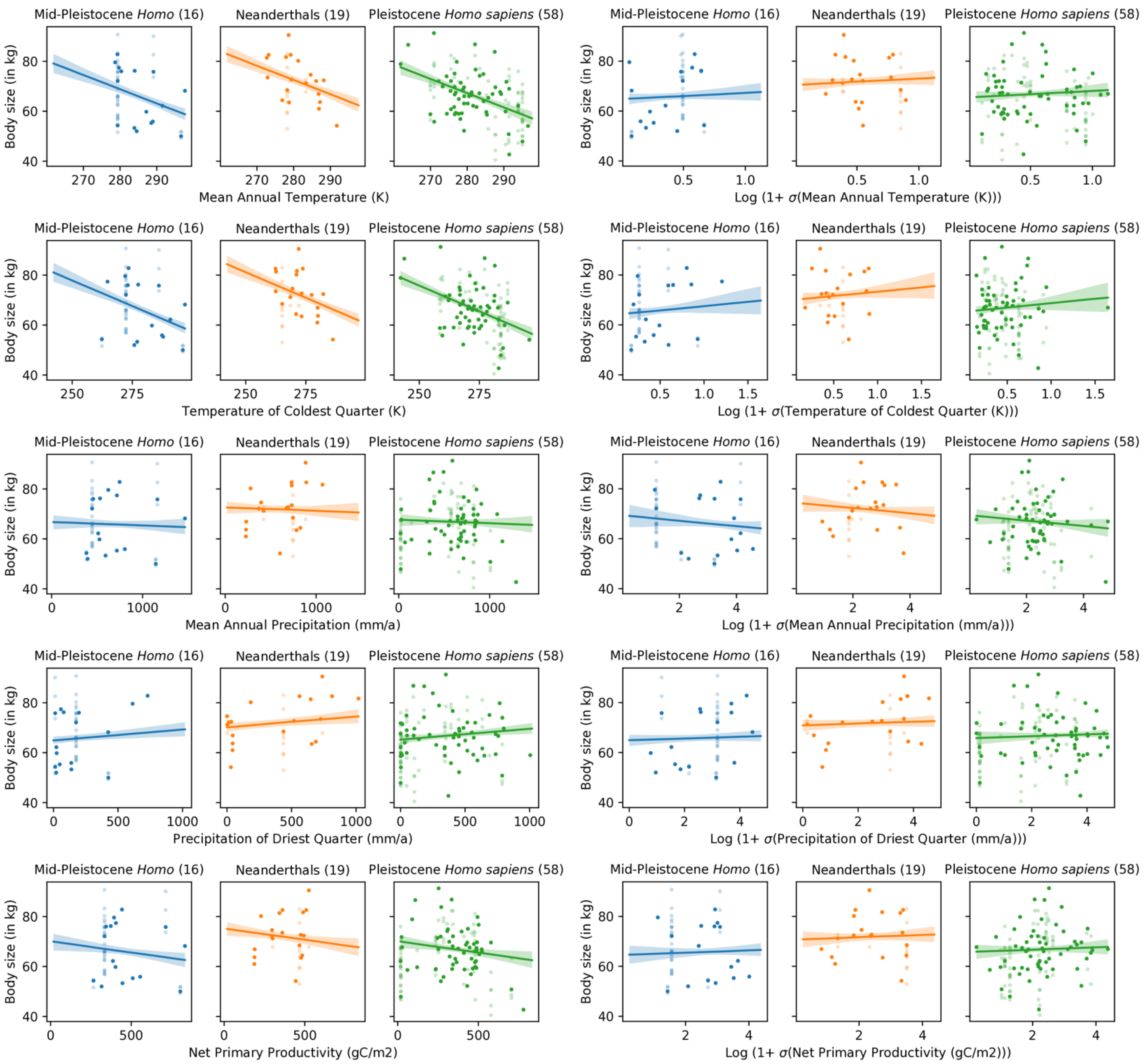

Supplementary Figure 3. Analysis of body size (log units) fossil data with the *LM-TC* model. Relationships based on *LM-TC* between body size from the fossil data sets and the ten climate variables based on the local climate reconstructions. The shaded band corresponds to the 95-percentile range (2.5-97.5%) of all linear regression lines that have been calculated for the 1000 randomized and thinned samples with the thick line in the centre corresponding to the median (50th-percentile). Each semi-transparent point represents a single fossil record, whereas the opaque points represent a record from a randomly thinned sub-sample. Numbers in brackets indicate the number of fossil records for each taxonomic unit in thinned subsamples.

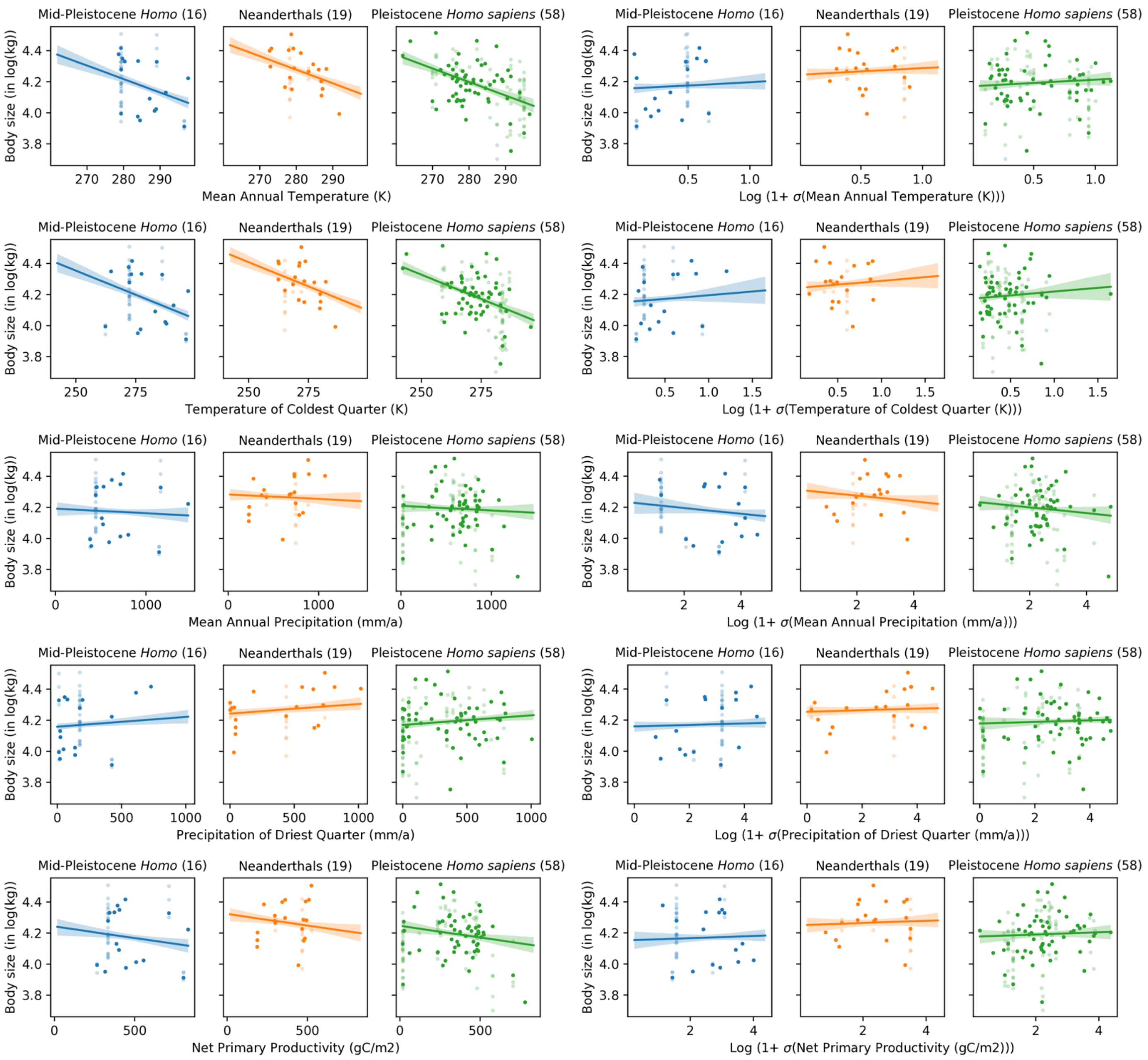

Supplementary Figure 4. Analysis of body size (natural units) fossil data with the *LM-T*C* model. Relationships based on *LM-T*C* between body size from the fossil data sets and the ten climate variables based on the local climate reconstructions. The shaded band corresponds to the 95-percentile range (2.5-97.5%) of all linear regression lines that have been calculated for the 1000 randomized and thinned samples with the thick line in the centre corresponding to the median (50th-percentile). Each semi-transparent point represents a single fossil record, whereas the opaque points represent a record from a randomly thinned sub-sample. Numbers in brackets indicate the number of fossil records for each taxonomic unit in thinned subsamples.

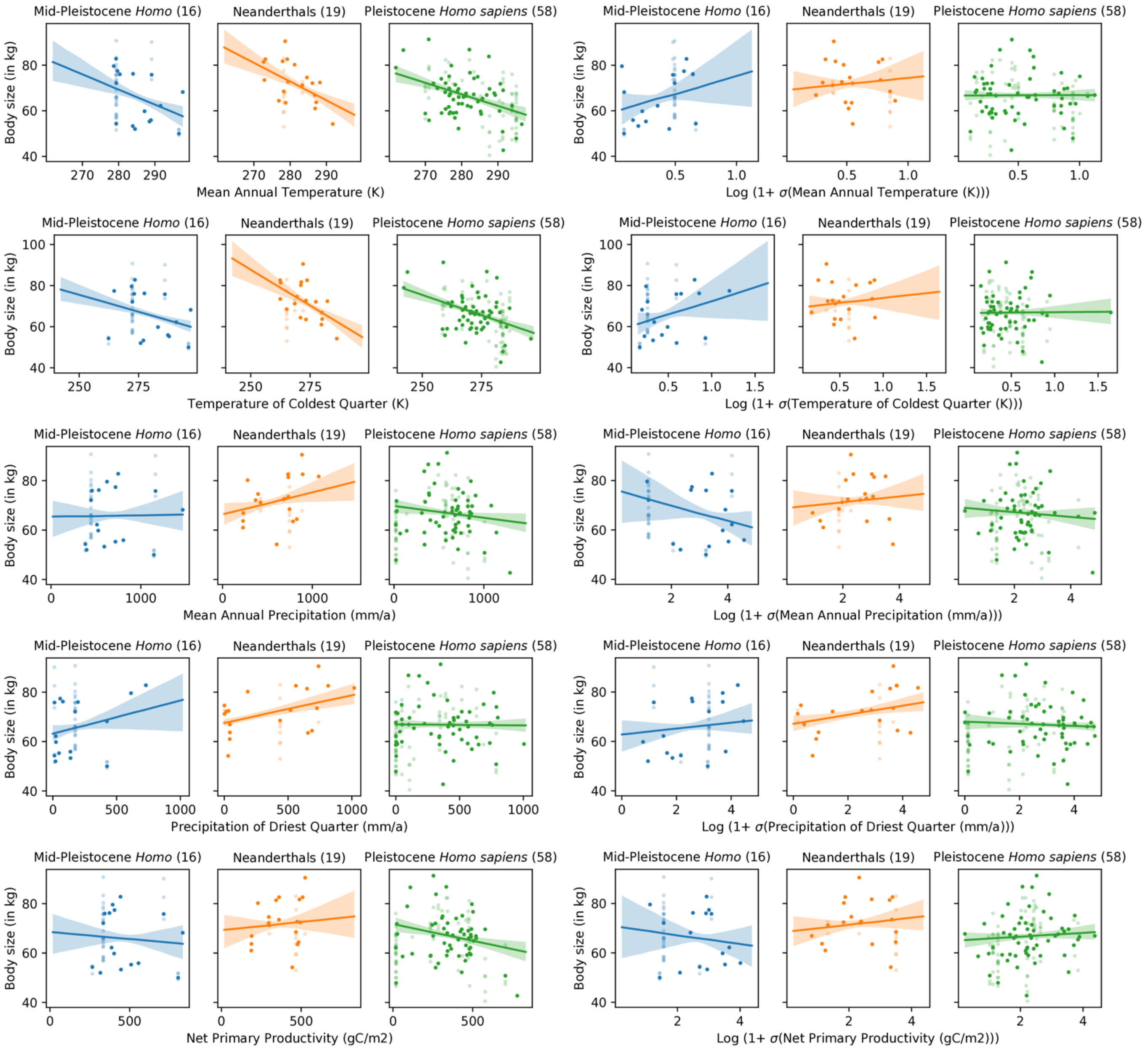

Supplementary Figure 5. Analysis of body size (log units) fossil data with the *LM-T*C* model. Relationships based on *LM-T*C* between body size from the fossil data sets and the ten climate variables based on the local climate reconstructions. The shaded band corresponds to the 95-percentile range (2.5-97.5%) of all linear regression lines that have been calculated for the 1000 randomized and thinned samples with the thick line in the centre corresponding to the median (50th-percentile). Each semi-transparent point represents a single fossil record, whereas the opaque points represent a record from a randomly thinned sub-sample. Numbers in brackets indicate the number of fossil records for each taxonomic unit in thinned subsamples.

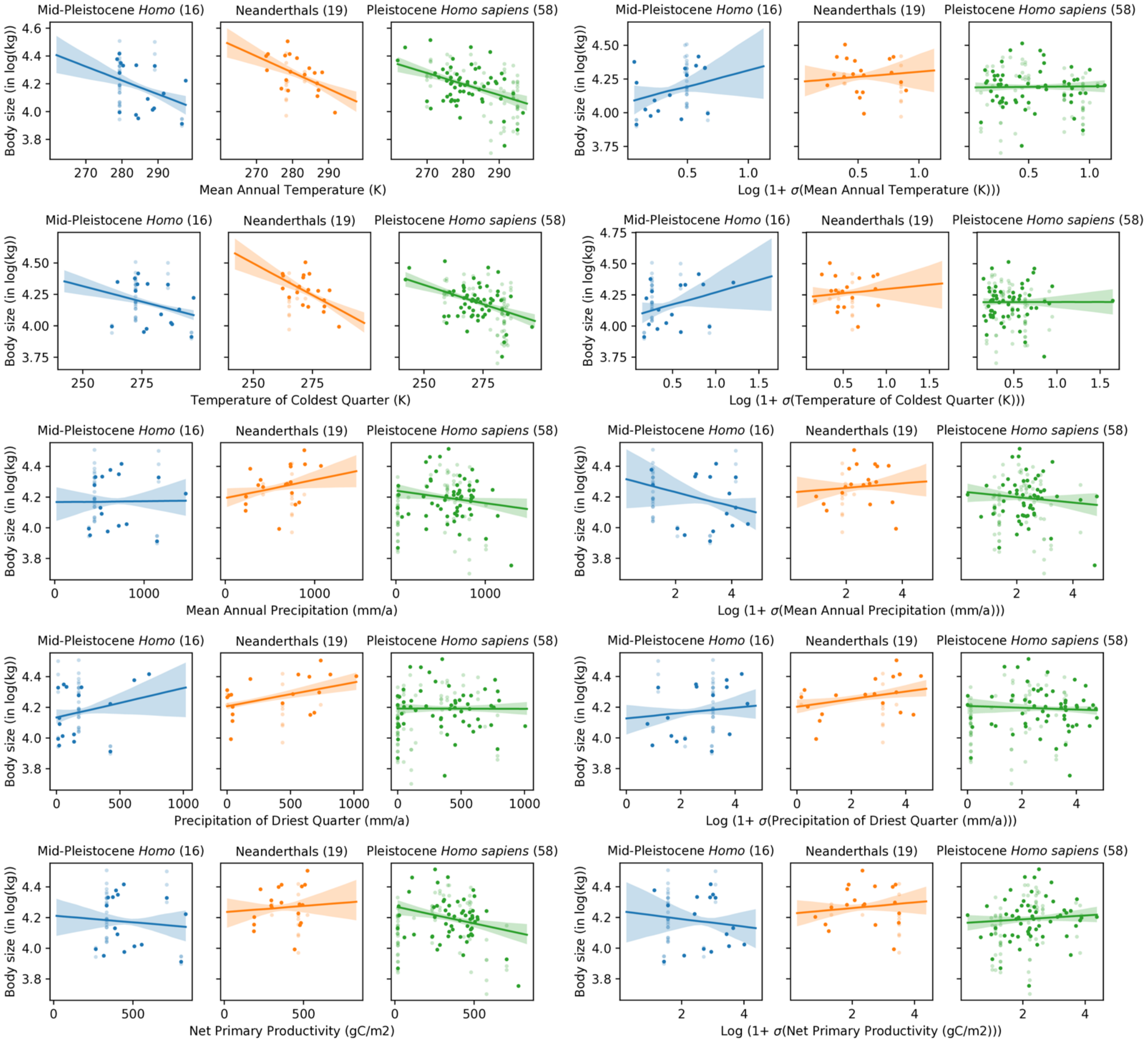

Supplementary Figure 6. Analysis of brain size (natural units) fossil data with the *LM-TC* model. Relationships based on *LM-TC* between brain size from the fossil data sets and the ten climate variables based on the local climate reconstructions. The shaded band corresponds to the 95-percentile range (2.5-97.5%) of all linear regression lines that have been calculated for the 1000 randomized and thinned samples with the thick line in the centre corresponding to the median (50th-percentile). Each semi-transparent point represents a single fossil record, whereas the opaque points represent a record from a randomly thinned sub-sample. Numbers in brackets indicate the number of fossil records for each taxonomic unit in thinned subsamples.

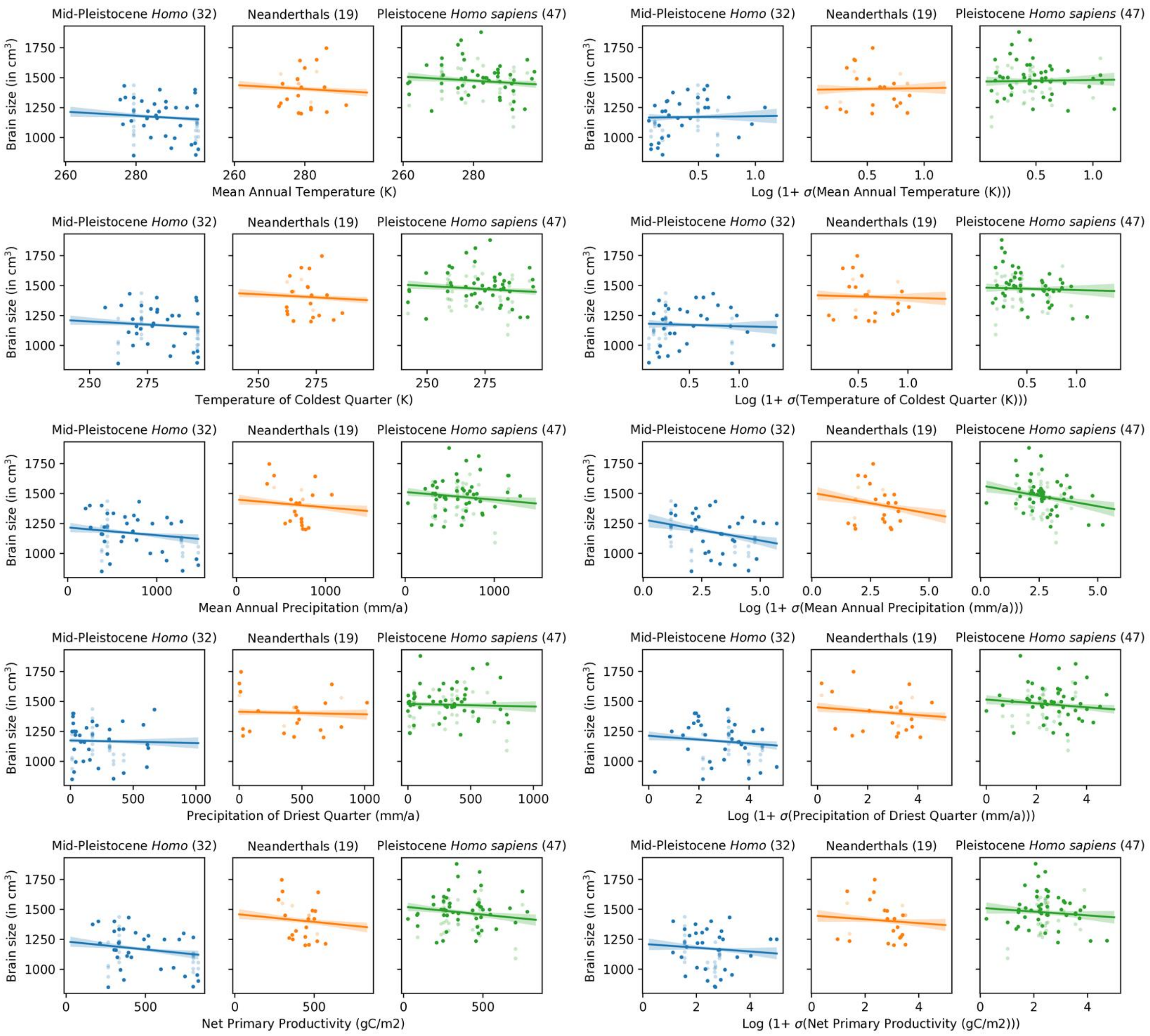

Supplementary Figure 7. Analysis of brain size (log units) fossil data with the *LM-TC* model. Relationships based on *LM-TC* between brain size from the fossil data sets and the ten climate variables based on the local climate reconstructions. The shaded band corresponds to the 95-percentile range (2.5-97.5%) of all linear regression lines that have been calculated for the 1000 randomized and thinned samples with the thick line in the centre corresponding to the median (50th-percentile). Each semi-transparent point represents a single fossil record, whereas the opaque points represent a record from a randomly thinned sub-sample. Numbers in brackets indicate the number of fossil records for each taxonomic unit in thinned subsamples.

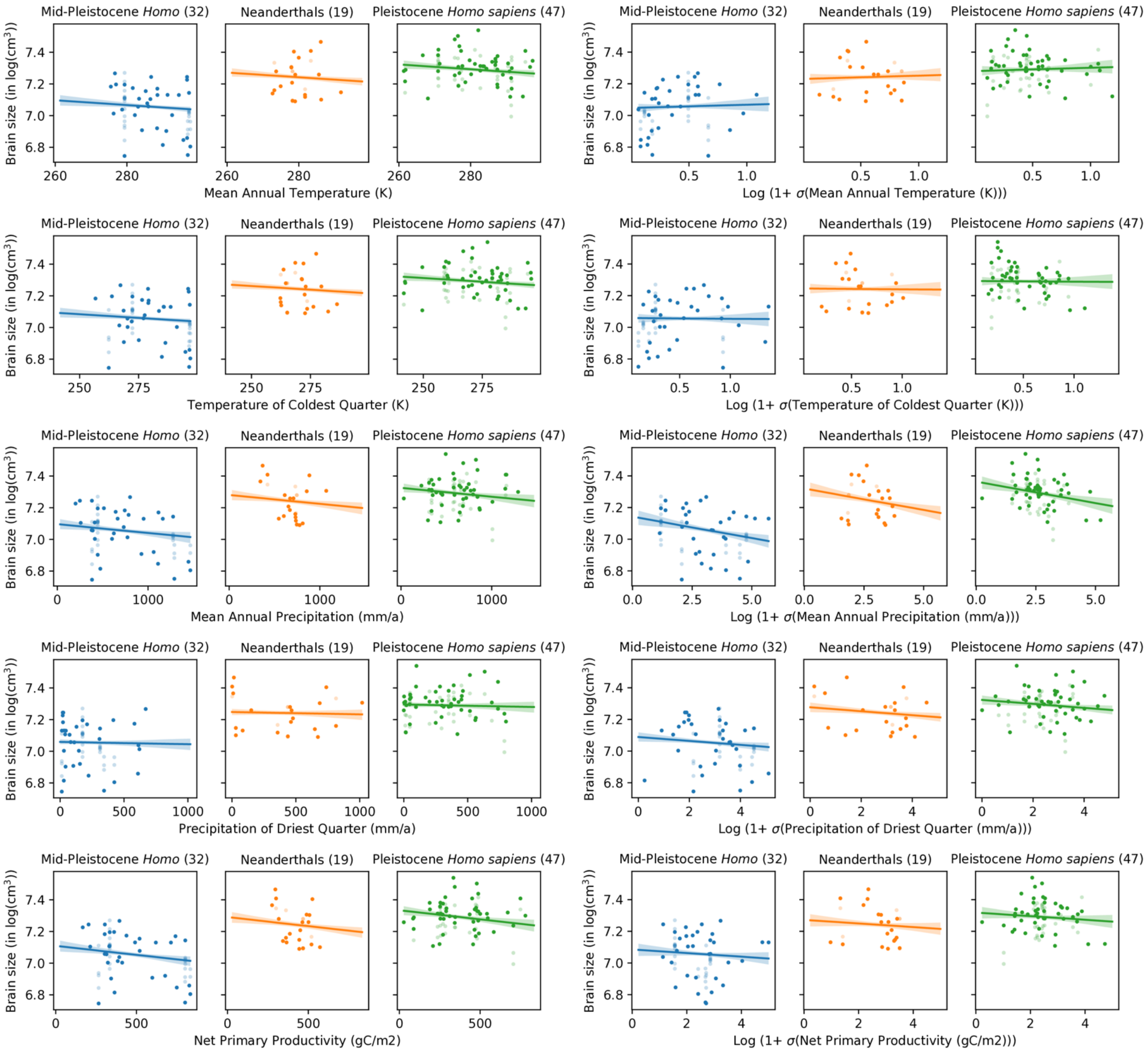

Supplementary Figure 8. Analysis of brain size (natural units) fossil data with the *LM-T*C* model. Relationships based on interaction models between brain size from the fossil data sets and the ten climate variables based on the local climate reconstructions. The shaded band corresponds to the 95-percentile range (2.5-97.5%) of all linear regression lines that have been calculated for the 1000 randomized and thinned samples with the thick line in the centre corresponding to the median (50th-percentile). Each semi-transparent point represents a single fossil record, whereas the opaque points represent a record from a randomly thinned sub-sample. Numbers in brackets indicate the number of fossil records for each taxonomic unit in thinned subsamples.

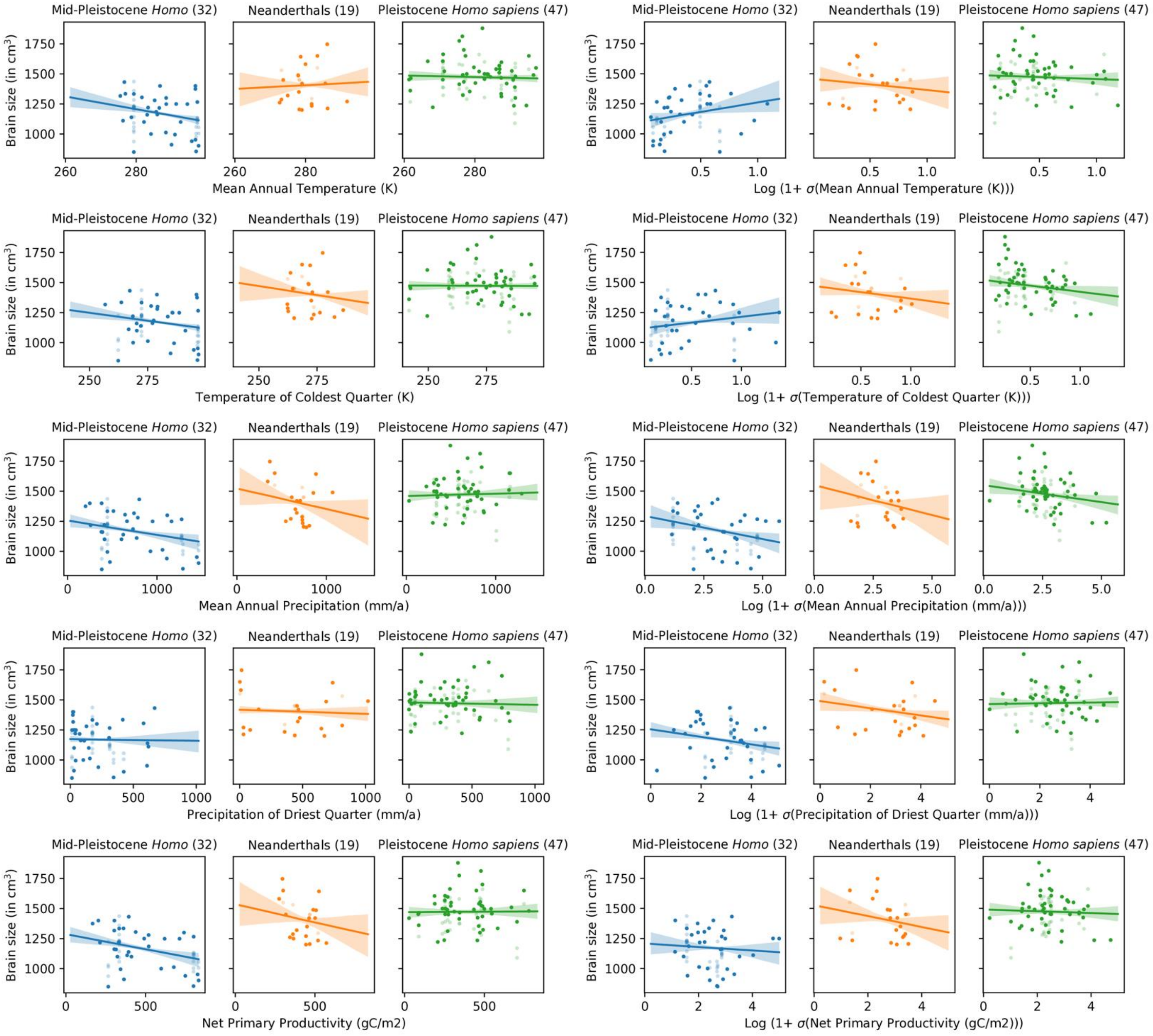

Supplementary Figure 9. Analysis of brain size (log units) fossil data with the *LM-T*C* model. Relationships based on interaction models between brain size from the fossil data sets and the ten climate variables based on the local climate reconstructions. The shaded band corresponds to the 95-percentile range (2.5-97.5%) of all linear regression lines that have been calculated for the 1000 randomized and thinned samples with the thick line in the centre corresponding to the median (50th-percentile). Each semi-transparent point represents a single fossil record, whereas the opaque points represent a record from a randomly thinned sub-sample. Numbers in brackets indicate the number of fossil records for each taxonomic unit in thinned subsamples.

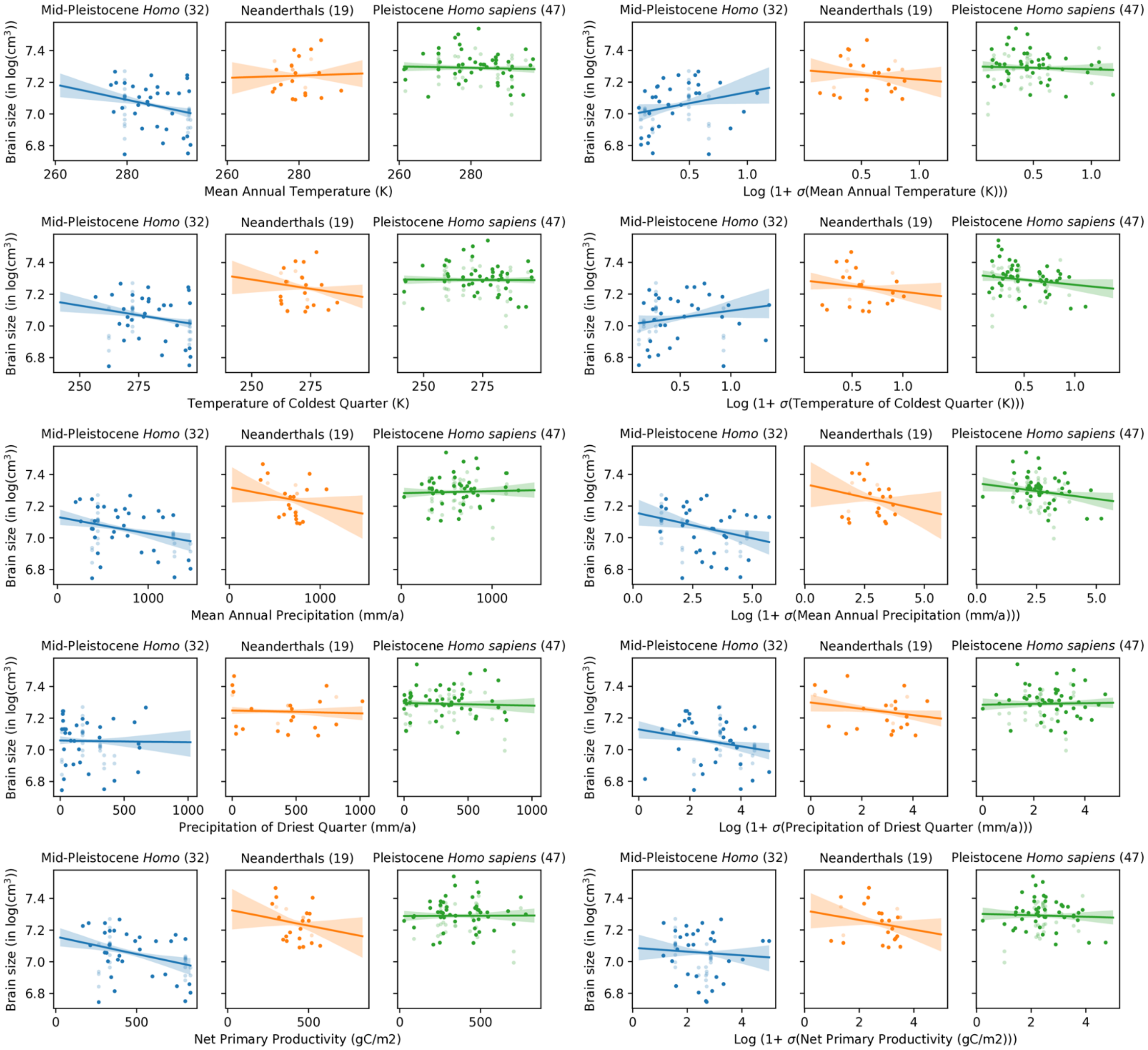

Supplementary Figure 10. Synthetic samples of body size as time series in comparison with the original fossil data (log units). Realizations of two synthetic body size data sets (orange and blue) for the three different relationship strengths between body size and the climate variable (*weak*, *medium*, and *strong*) and how they compare to the actual fossil data.

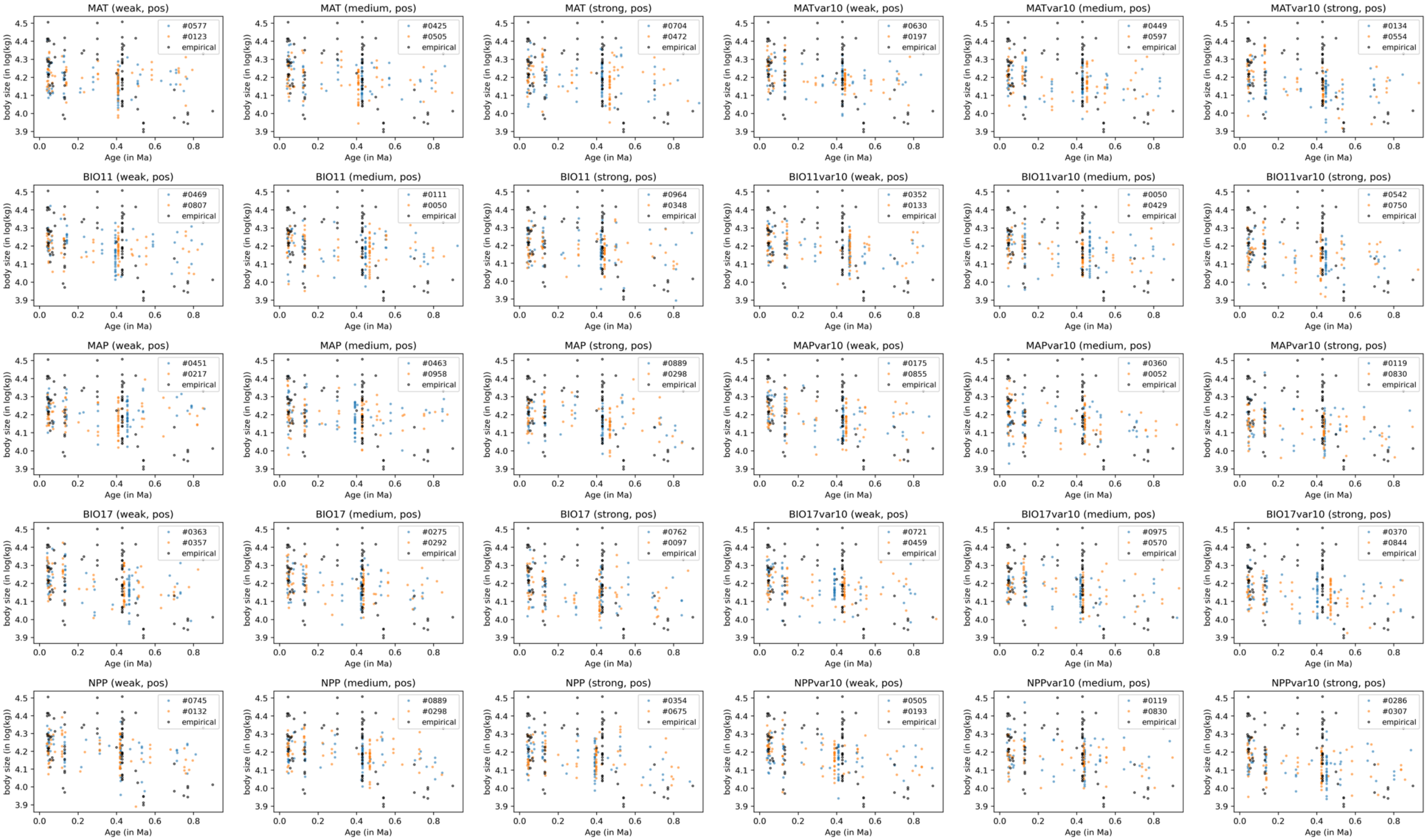

Supplementary Figure 11. Synthetic samples of brain size as time series in comparison with the original fossil data (log units). Realizations of two synthetic brain size data sets (orange and blue) for the three different relationship strengths between brain size and the climate variable (*weak*, *medium*, and *strong*) and how they compare to the actual fossil data.

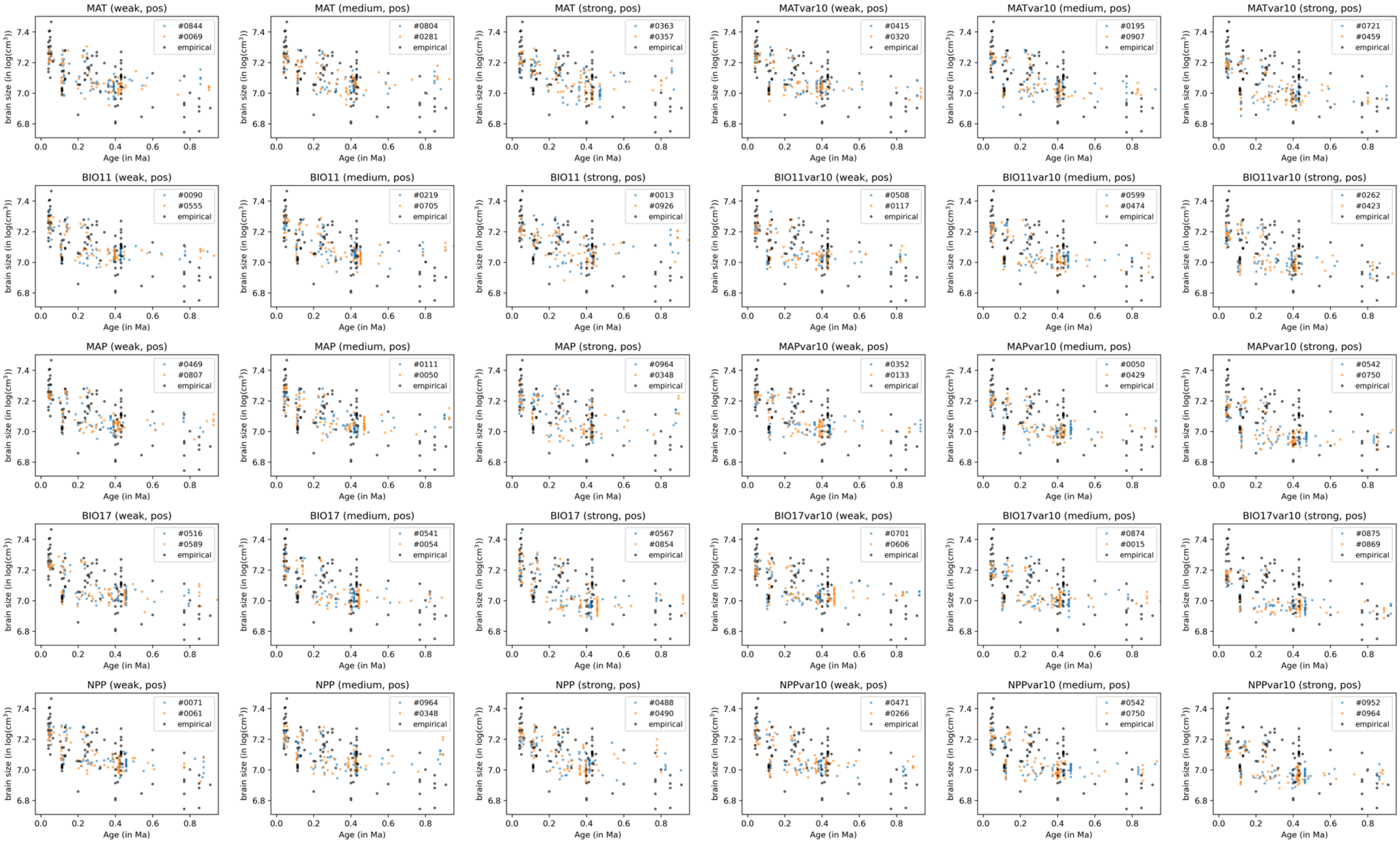

Supplement: Supplementary file 1 — Supplementary Information [file 41467_2021_24290_MOESM1_ESM.pdf]
