# Supplementary material for: Different environmental variables predict body and brain size evolution in Homo
Source: Nat Commun. 2021 Jul 8;12:4116. doi: 10.1038/s41467-021-24290-7 (PMC8266824; doi:10.1038/s41467-021-24290-7)
Supplement: Supplementary file 3 — Description of Additional Supplementary Files [file 41467_2021_24290_MOESM3_ESM.pdf]

Description of Additional Supplementary Files

File Name: Supplementary Data 1
Description: The database of fossil body size of *Homo* is attached as separate Excel file and includes information on: Unique specimen ID, Age in million years (Ma), Geographical X-coordinate (longitude), Geographical Y-coordinate (latitude), Taxonomic assessment, Body size estimate (body mass, in kg), Age group, Source of data (see also Will et al. 2017 for more data references and the original data publication), Dating method, Dating source and comment.

File Name: Supplementary Data 2
Description: The database of fossil brain size of *Homo* is attached as separate Excel file and includes information on: Unique specimen ID, Age in million years (Ma), Geographical X-coordinate (longitude), Geographical Y-coordinate (latitude), Taxonomic assessment, Brain size estimate (endocranial volume, in cm^3), Age group, Source of data, Dating method, Dating source and comment.
