# Supplementary material for: Different environmental variables predict body and brain size evolution in Homo
Source: Nat Commun. 2021 Jul 8;12:4116. doi: 10.1038/s41467-021-24290-7 (PMC8266824; doi:10.1038/s41467-021-24290-7)
Supplement: Supplementary file 6 — Reporting Summary [file 41467_2021_24290_MOESM6_ESM.pdf]

nature research

Corresponding author(s): Manuel Will

Last updated by author(s): May 31, 2021

Reporting Summary

Nature Research wishes to improve the reproducibility of the work that we publish. This form provides structure for consistency and transparency in reporting. For further information on Nature Research policies, see our Editorial Policies and the Editorial Policy Checklist.

Statistics

For all statistical analyses, confirm that the following items are present in the figure legend, table legend, main text, or Methods section.

n/a	Confirmed	
☐	☒	The exact sample size (*n*) for each experimental group/condition, given as a discrete number and unit of measurement
☒	☐	A statement on whether measurements were taken from distinct samples or whether the same sample was measured repeatedly
☐	☒	The statistical test(s) used AND whether they are one- or two-sided<br>*Only common tests should be described solely by name; describe more complex techniques in the Methods section.*
☒	☐	A description of all covariates tested
☐	☒	A description of any assumptions or corrections, such as tests of normality and adjustment for multiple comparisons
☐	☒	A full description of the statistical parameters including central tendency (e.g. means) or other basic estimates (e.g. regression coefficient) AND variation (e.g. standard deviation) or associated estimates of uncertainty (e.g. confidence intervals)
☒	☐	For null hypothesis testing, the test statistic (e.g. F, t, r) with confidence intervals, effect sizes, degrees of freedom and P value noted<br>*Give P values as exact values whenever suitable.*
☒	☐	For Bayesian analysis, information on the choice of priors and Markov chain Monte Carlo settings
☒	☐	For hierarchical and complex designs, identification of the appropriate level for tests and full reporting of outcomes
☐	☒	Estimates of effect sizes (e.g. Cohen's d, Pearson's r), indicating how they were calculated

Our web collection on statistics for biologists contains articles on many of the points above.

Software and code

Policy information about availability of computer code

Data collection	Climate data derived from the global climate model emulator (GCMET) and can be found here: EarthArXiv. doi:10.31223/osf.io/d5hfx (v2, 2019) and here doi:10.17605/OSF.IO/SMYAC. No specific software was used for data collection of fossil body and brain sizes.
Data analysis	Statistical tests were undertaken in Python version 3.8.5 using the following Python packages: statsmodels 0.12 (for linear models), pandas 1.1.3 (for dataframes, reading/writing CSV/Excel files), netCDF4 1.5.3 (reading NetCDF files), matplotlib 3.3.2 (for plotting), numpy 1.19.2 (numerics). The source code used to run the analyses can be found uploaded here: doi:10.17605/OSF.IO/SMYAC

For manuscripts utilizing custom algorithms or software that are central to the research but not yet described in published literature, software must be made available to editors and reviewers. We strongly encourage code deposition in a community repository (e.g. GitHub). See the Nature Research guidelines for submitting code & software for further information.

Data

Policy information about availability of data

All manuscripts must include a data availability statement. This statement should provide the following information, where applicable:

- Accession codes, unique identifiers, or web links for publicly available datasets
- A list of figures that have associated raw data
- A description of any restrictions on data availability

Data on fossil specimens were collected from the literature (body and brain sizes) and our own previous research. This data and all sources for each data point are provided in Supplementary Data 1 & 2. Information on modern variation of brain size derives from the William W. Howells Craniometric Data Set accessible via http://volweb.utk.edu/~auerbach/HOWL.htm. All data generated or analyzed during this study are included in this published article and its supplementary information files. The climate data to run all analyses in this paper can be accessed via doi:10.17605/OSF.IO/SMYAC

Field-specific reporting

Please select the one below that is the best fit for your research. If you are not sure, read the appropriate sections before making your selection.

☐ Life sciences ☐ Behavioural & social sciences ☒ Ecological, evolutionary & environmental sciences

For a reference copy of the document with all sections, see nature.com/documents/nr-reporting-summary-flat.pdf

Ecological, evolutionary & environmental sciences study design

All studies must disclose on these points even when the disclosure is negative.

Study description	The study tests the influence of environmental factors on the evolution of body and brain size in the genus Homo over the last ~1 Ma. We formally test the relationship between body/brain size and local climatic variables in accordance with formalized environmental hypotheses using three different linear models. Given the many biases and uncertainties of hominin fossil data, for each environmental hypothesis, we first estimate the power of such a dataset as ours to detect relationships of different environmental effects via the generation of 1000 synthetic datasets for all climate variable associations with body and brain size. In a second step, we test the four environmental hypotheses for body and brain size among the Homo lineage with the real fossil datasets via three different linear models.
Research sample	Data on fossil specimens were collected from the literature (body and brain sizes) and our own previous research. All sources for each data point are provided in Supplementary Data 1 & 2. Our body (n=204) and brain size (n=166) estimates come from individual hominin fossils distributed over the Old World and ranging from ca. 1.0-0.01 Ma. We divide this dataset into three taxonomic units: Mid-Pleistocene Homo, Homo neanderthalensis and Pleistocene Homo sapiens. The environmental information for each individual data point comes from a climate emulator (GCMET) that takes into account long-term, glacial-interglacial climate variation, caused by changes in the Earth's orbit around the sun (Milankovitch cycles) and in greenhouse gases, such as CO2.
Sampling strategy	We endeavoured to collect all published body and brain size estimates for adult Homo available from the literature distributed over the Old World and ranging from ca. 1.0-0.01 Ma. As a result, this study rests on the largest databases of body and brain size estimates collected of this time frame so far. No specific (sub-)sampling strategy was applied.
Data collection	Body and brain size estimates were collected from the literature and our own published data. See Supplementary Data 1 & 2 for the sources of each data point. The climate data derived from the GCMET by M. Krapp et al. EarthArXiv. doi:10.31223/osf.io/d5hfx (v2, 2019).
Timing and spatial scale	Body and brain size data from the Old World dating to between 1.0-0.01 Ma and assigned to the genus Homo were included with a few exceptions (see below).
Data exclusions	Brain and body size estimates from Homo floresiensis and Homo naledi were excluded from this analysis as we cannot use these taxonomic units to study the effects of environmental variables on body/brain size. H. floresiensis as a taxonomic unit has only produced one body and brain size estimate from a single site and age and so does not feature any environmental variation to study. Further, Homo floresiensis has not been assigned to any of the taxonomic units we used here. Principally the same applies to Homo naledi which is so-far known from one time slice and one site, though several body and brain size estimates exist. These exclusion critera were pre-established as part of the initial study design.
Reproducibility	We provide all sources of raw data and codes of analyses so that the study can be reproduced.
Randomization	Randomization was used in the analysis of the synthetic and fossil datasets. To avoid a few oversampled fossil sites that contain multiple hominin specimens with the same age driving the results, each synthetic dataset used in the power analysis was randomly thinned by only retaining one specimen for any given location-time combination. This process was repeated to generate 1000 randomly thinned versions of each of the datasets. The same thinning was applied for the analysis of the real hominin fossil datasets.
Blinding	Blinding does not apply to this study as it is based on already established datasets.

Did the study involve field work? ☐ Yes ☒ No

Reporting for specific materials, systems and methods

We require information from authors about some types of materials, experimental systems and methods used in many studies. Here, indicate whether each material, system or method listed is relevant to your study. If you are not sure if a list item applies to your research, read the appropriate section before selecting a response.

Materials & experimental systems

n/a	Involved in the study
☒	☐ Antibodies
☒	☐ Eukaryotic cell lines
☐	☒ Palaeontology and archaeology
☒	☐ Animals and other organisms
☒	☐ Human research participants
☒	☐ Clinical data
☒	☐ Dual use research of concern

Methods

n/a	Involved in the study
☒	☐ ChIP-seq
☒	☐ Flow cytometry
☒	☐ MRI-based neuroimaging

Palaeontology and Archaeology

Specimen provenance	No permits were required for conducting this meta-analysis of hominini fossil brain and body size estimates. No hominin fossils were collected or directly accessed for this study.
Specimen deposition	This category does not apply to this meta-study. Geographical origin (latitude and longitude) are provided for each hominin fossil specimen part of this study in Supplementary Data 1 & 2.
Dating methods	Dating methods and dating uncertainty is provided for each hominin fossil specimen included in this study in Supplementary Data 1 & 2. This information was taken from the literature and no new datings were performed as part of this study.

☒ Tick this box to confirm that the raw and calibrated dates are available in the paper or in Supplementary Information.

Ethics oversight	No ethical approval or guidance was needed as this was a meta-study of hominini fossil brain and body size estimates, collecting data from the literatur or our own previous research.

Note that full information on the approval of the study protocol must also be provided in the manuscript.
